# Prediction of sinus rhythm maintenance following DC-cardioversion of persistent atrial fibrillation – the role of atrial cycle length

**DOI:** 10.1186/1471-2261-6-11

**Published:** 2006-03-13

**Authors:** Carl J Meurling, Anders Roijer, Johan EP Waktare, Fredrik Holmqvist, Carl J Lindholm, Max P Ingemansson, Jonas Carlson, Martin Stridh, Leif Sörnmo, S Bertil Olsson

**Affiliations:** 1 Department of Cardiology, Lund University Hospital, SE-221 85 Lund, Sweden; 2 The Cardiothoracic Centre, Liverpool, UK; 3 Department of Applied Electronics, Lund Institute of Technology, Lund, Sweden

## Abstract

**Background:**

Atrial electrical remodeling has been shown to influence the outcome the outcome following cardioversion of atrial fibrillation (AF) in experimental studies.

The aim of the present study was to find out whether a non-invasively measured atrial fibrillatory cycle length, alone or in combination with other non-invasive parameters, could predict sinus rhythm maintenance after cardioversion of AF.

**Methods:**

Dominant atrial cycle length (DACL), a previously validated non-invasive index of atrial refractoriness, was measured from lead V1 and a unipolar oesophageal lead prior to cardioversion in 37 patients with persistent AF undergoing their first cardioversion.

**Results:**

32 patients were successfully cardioverted to sinus rhythm. The mean DACL in the 22 patients who suffered recurrence of AF within 6 weeks was 152 ± 15 ms (V1) and 147 ± 14 ms (oesophagus) compared to 155 ± 17 ms (V1) and 151 ± 18 ms (oesophagus) in those maintaining sinus rhythm (NS). Left atrial diameter was 48 ± 4 mm and 44 ± 7 mm respectively (NS). The optimal parameter predicting maintenance of sinus rhythm after 6 weeks appeared to be the ratio of the lowest dominant atrial cycle length (oesophageal lead or V1) to left atrial diameter. This ratio was significantly higher in patients remaining in sinus rhythm (3.4 ± 0.6 vs. 3.1 ± 0.4 ms/mm respectively, p = 0.04).

**Conclusion:**

In this study neither an index of atrial refractory period nor left atrial diameter alone were predictors of AF recurrence within the 6 weeks of follow-up. The ratio of the two (combining electrophysiological and anatomical measurements) only slightly improve the identification of patients at high risk of recurrence of persistent AF. Consequently, other ways to asses electrical remodeling and / or other variables besides electrical remodeling are involved in determining the outcome following cardioversion.

## Background

Once atrial fibrillation (AF) has been present for more than a few days, the optimal method for restoring sinus rhythm is DC-cardioversion[1,2]. Unfortunately, only about 25% of the patients remain in sinus rhythm at one year post-cardioversion, with the proportion rising to approximately half of patients if pharmacoprophylaxis is employed [3-5]. Most patients who relapse to AF the first year do so within a few weeks of cardioversion[6,7].

A number of clinical and demographic features have been suggested to be helpful in predicting successful cardioversion and long term maintenance of sinus rhythm post-cardioversion[1-5,8]. Reports investigating the effects of prolonged tachycardia on atrial electrophysiology have found that high frequency depolarisation in the atria lead to electrophysiological changes such as shortening of the atrial refractory period, which promote self-perpetuation of the arrhythmia [9-11]. This phenomenon, termed atrial electrical remodeling, is at least partly reversible following arrhythmia termination in animal models[9,12,13] but data in man is conflicting[14,15]. Furthermore, inducibility of AF is high during the period of recovery and correlates to the length of the refractory period[9,10,12,15], which may explain the high propensity of AF to relapse early post-cardioversion. The role of the length of atrial refractory period in patients with persistent AF (i.e. already established electrical remodeling) in predicting outcome of DC-cardioversion is unclear. A recent study addressed the question with positive results, but its complex patient selection makes it difficult to infer the results to clinical practice[16].

Since both electrophysiological and morphological factors may influence the outcome of cardioversion, the aim of this study was to evaluate whether an index of atrial refractoriness during AF, a probable marker of the degree of fibrillation-induced remodeling, alone or in combination with anatomical atrial parameters could predict outcome of elective DC-cardioversion of persistent AF.

## Methods

### Study population

Patients referred to our department with persistent AF undergoing their first elective DC conversion were screened for possible recruitment to the study. Exclusion criteria were overt heart failure and coronary heart disease, hyperthyroidism or ongoing treatment with Vaughan Williams class I or III anti-arrhythmic drugs.

All patients gave informed consent. The study complies with the Declaration of Helsinki and was approved by the local Ethics Committee, Faculty of Medicine, Lund University, Lund, Sweden.

### Frequency analysis of fibrillatory ECG

Frequency analysis of fibrillatory ECG (FAF-ECG) is a validated, non-invasive method for estimating atrial fibrillatory rate by computerized processing of a surface recorded ECG signal [17-19]. It involves the recording of high resolution ECG, computerized identification, templating and removal of ventricular components of the ECG to leave a pure atrial recording, and finally frequency domain based analysis of this atrial recording.

A standard 12-lead ECG was acquired using a custom made optically isolated PC card (Siemens Elema AB, Solna, Sweden). The electrode position configuration is conventional with the exception that the C6 chest lead is connected to an oesophageal electrode (Medtronic 6992A, Medtronic Inc., Minneapolis, Min. USA) to give a unipolar oesophageal electrogram. The digital signal (1 kHz sampling, 16 bit analogue-to-digital conversion, 0.6 μV resolution) was transferred to a personal computer, where the data was written to a file for subsequent off-line processing.

Cancellation of the QRST is performed and the residual signal subjected to frequency domain analysis using fast Fourier transformation. The frequency peak in the 3 to 12 Hz band with the highest amplitude is automatically chosen by the analytical software. This value is converted to a cycle length termed the dominant atrial cycle length (DACL), which has been shown to be an index of atrial refractoriness [20-22]. In this study, lead V1 and the unipolar oesophageal lead were analysed. Previous studies have shown that the V1 lead correlates well with the spatial mean of intracardiac recordings from the right atrial free wall[23]. The oesophageal lead has more extensive influences, correlating to posterior left and right atria as well as the interatrial septal intracardiac signals[23]. Since there are some evidence that the left atrium is more involved in the initiation mechanism of AF[24], there are some theoretical advantages using an oesophageal lead.

### Cardioversion

FAF-ECG was acquired after 15 minutes supine rest, 30–60 minutes before elective DC cardioversion. DC-conversion was performed with one electrode paddle in a right parasternal position and the other at the cardiac apex (lateral thorax in the mid-axillary line), with initial shock energy of 200 J. If unsuccessful, another shock of 200 J, and finally a shock of 360 J was given. Successful DC cardioversion was defined as electrocardiographic documentation of abolition of AF with at least two sinus beats seen.

### Follow-up

All patients in sinus rhythm underwent 12-lead ECG prior to hospital discharge (4 hours), and at 1, 3 and 6 weeks post cardioversion. Cardioactive medications were left unchanged for the duration of the study.

### Statistics

Values are expressed as mean values ± standard deviation, unless otherwise specified. Duration of sinus rhythm is expressed as median and range. The Mann-Whitney U test and Fisher's exact test was used to determine whether there was any significant difference between the groups. Spearman's rank correlation coefficient was calculated to determine whether there was any correlation between DACL and left atrial diameter. A p < 0.05 was considered statistically significant. Statistical analyses were performed using StatView for Windows, version 4.5 (Abacus Concepts, Inc., Berkeley, CA, USA).

## Results

### Study population

The study population compromised 37 outpatients (27 male). Relevant co-morbidity included hypertension (8 patients), moderate valvular heart disease (1 patient each with aortic stenosis and mitral regurgitation) and chronic airflow obstruction (4 patients). Cardioactive pharmacotherapy included β-blockers (4 patients), calcium channel antagonists (3 patients), ACE-inhibitors (3 patients) and digoxin (14 patients). Mean age was 69 ± 6 years (range 58 to 84 years) and the median AF duration was 5 months (range 1 to 21 months). Echocardiographic mean left atrial diameter (anterior-posterior dimension in the parasternal view) was 47 ± 5 mm (range 35 to 57 mm), and the mean left ventricular ejection fraction was 51 ± 9 % (range 20 to = 55%). The clinical characteristics of the subjects are summarised in Table 1.

**Table 1:** Clinical characteristics

AGE	< 65 yrs	65–74 yrs	≥ 75 yrs
	11	19	7
WEIGTH	< 75 kg	75–89 kg	≥ 90 kg
	6	19	11
AF-duration	< 4 months	4–12 months	≥ 12 months
	13	20	4
EF	< 40 %	40–54 %	≥ 55 %
	5	6	26
LA-diameter	< 40 mm	40–49 mm	≥ 50 mm
	3	22	12

Summary of the clinical and echocardiographic characteristics of the 37 patients recruited to the study. Weight data is missing for one patient.

Cardioversion restored sinus rhythm in 32 of the 37 patients in the study, but at 6 weeks only 10 patients remained in sinus rhythm (Figure 2). Analysable frequency spectra ("FAF-ECG's") were obtained in 34 patients (one of these only had a satisfactory spectrum in V1, leaving 33 patients for analysis of oesophageal lead data).

**Figure 1:**
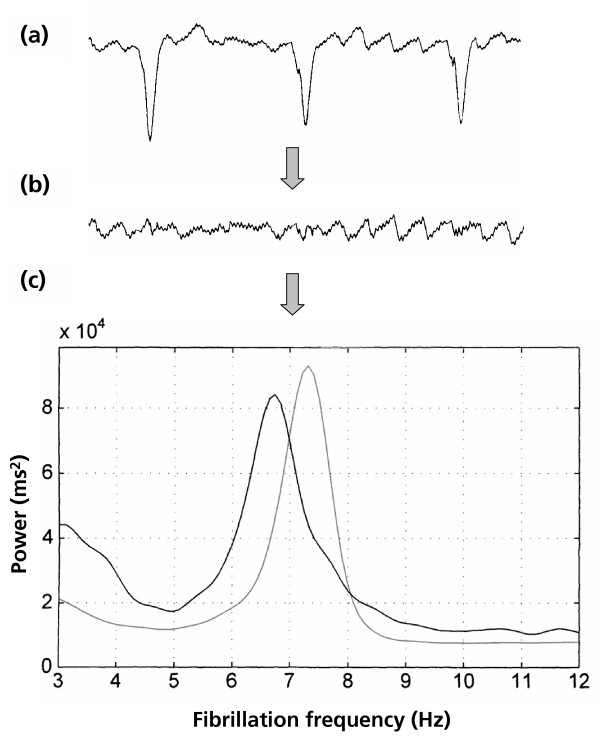
**Illustration of the FAF-ECG method. **Illustration of the process by which DACL is calculated, and explanation of the additional parameters which were tested in this study. (a) The digital ECG is analysed and the QRST identified and templated. (b) The template is then subtracted from the raw ECG to give a signal representing the atrial fibrillation alone. (c) The frequency at which the peak is seen is expressed as a cycle length to give the DACL. The DACL recorded from lead V1 (black line) is longer (i.e. fibrillation frequency lower) than that in oesophageal lead (grey line) in this patient, which is the most common finding.

**Figure 2:**
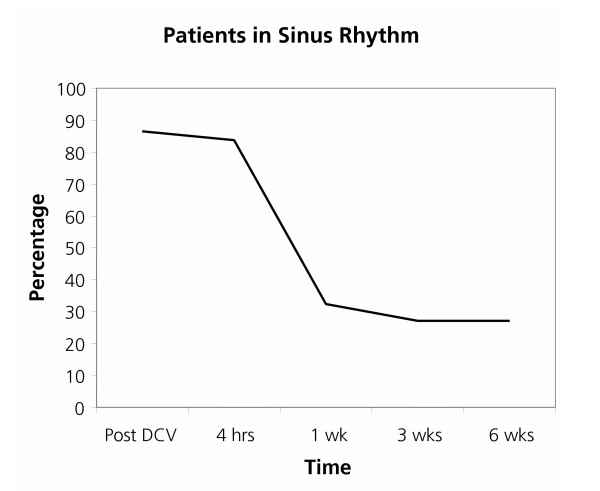
**Sinus rhythm maintenance following cardioversion. **An illustration of sinus rhythm maintenance in this study. Note that the abscissa is a non-linear scale due to the available follow-up data.

Of the other patient characteristics studied (i.e. gender, age, atrial fibrillation duration, and presence of underlying heart disease) and echocardiographic parameters measured (left atrial diameter and ejection fraction), none showed a definite relationship with conversion rate. There was a trend for those with a primary failure of cardioversion to be heavier (98 ± 30 kg vs. 85 ± 12 kg, NS) and for their BMI to be higher (31 ± 9 vs. 28 ± 4, NS).

**Figure 3:**
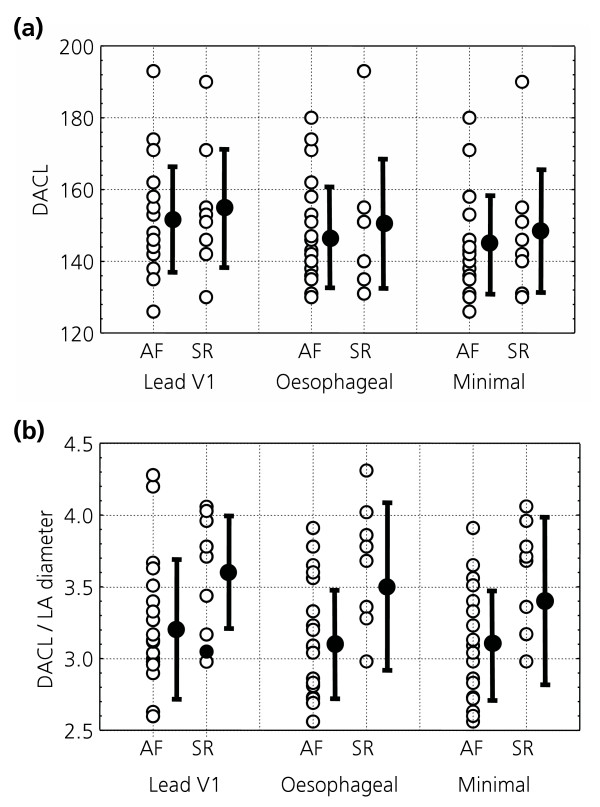
**Sinus rhythm maintenance following cardioversion. **(a) DACL, and (b) the ratio of DACL to left atrial diameter according to outcome. AF = patients in atrial fibrillation at 6 weeks after an initially successful cardioversion; SR = those in sinus rhythm; DACL = dominating atrial cycle length; LA = left atrial diameter; V1 = lead V1; Oes = unipolar oesophageal lead; Minimum = minimum DACL of the values in V1 and oesophagus. Solid circles represent the mean and error bars are ± 1SD.

Patients who remained in sinus rhythm tended to have shorter atrial fibrillation duration, a smaller left atrial diameter (Figure 3a) and a higher ejection fraction, but none of these variables alone reached statistical significance and other features such as age and body weight did not appear to influence atrial fibrillation recurrence (table 2).

**Table 2:** Measured parameters

	**SR**	**AF**	**p-value**
***Clinical***			
Male	9/10	13/22	0.11*
Age (years)	70 (5)	68 (7)	0.41
Weight (kg)	82 (9)	86 (13)	0.69
BMI (kg/m2)	27 (3)	29 (5)	0.23
AF Duration (months)	4 (2)	6 (2)	0.13
Lone AF	7/10	16/22	1.00*
***Anatomic***			
Ejection Fraction (%)	54 (2)	49 (10)	0.14
LA Diameter (mm)	44 (7)	48 (4)	0.08
***Electrophysiologic***			
DACL V1	155 (17)	152 (15)	0.69
DACL Oes	151 (18)	147 (14)	0.59
Shortest DACL	148 (18)	145 (13)	0.59
***Pre-specified combined parameters***			
DACL V1/LA	3.6 (0.4)	3.2 (0.5)	0.05
DACL Oes/LA	3.5 (0.6)	3.1 (0.4)	0.05
Shortest DACL/LA	3.4 (0.6)	3.1 (0.4)	0.04

Discriminatory power of tested parameters. These are listed grouped according to their demographic, anatomical or electrical nature. Some combined parameters were pre-specified and these are listed separate from other additional parameters. Key: BMI = body mass index; LA = left atrial diameter; DACL = dominating atrial cycle length; V1 = V1 lead; Oes = oesophageal lead; SR = sinus rhythm; AF = atrial fibrillation. Fischer exact test used to calculate p-values marked '*'. MannWhitney test used for all others.

### Prediction of successful cardioversion and AF recurrence by DACL

Patients successfully restored to sinus rhythm had a mean DACL in lead V1 of 153 ± 15 ms v 158 ± 21 ms in the patients who had a primary failure of cardioversion (NS). Comparable data for the oesophageal lead was 148 ± 15 ms vs. 151 ± 13 ms (NS).

Among patients who had been cardioverted, DACL was non-significantly longer in those who remained in sinus rhythm at 6 weeks (155 ± 17 ms in V1, 151 ± 18 ms in the oesophageal lead) than those who had relapsed to AF by this point (152 ± 15 ms in V1, 147 ± 14 ms in the oesophageal lead, p = NS for both, Figure 3b). Utilising the shortest DACL value from either lead did not improve discriminatory power (NS, table 2). DACL did not correlate with left atrial diameter, with a Spearman R = 0.25 for DACL from lead V1 (NS) and R = -0.04 from the oesophageal lead (NS).

### Combined electrophysiological and anatomical parameters to predict AF recurrence

**Figure 4:**
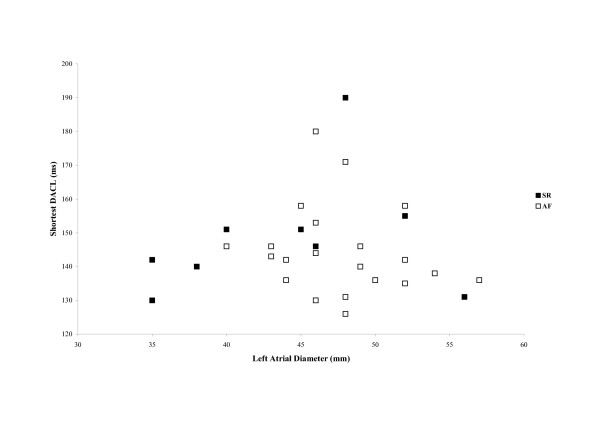
**Shortest DACL and Left atrial diameter. **Shortest DACL plotted against left atrial diameter. AF = atrial fibrillation; SR = sinus rhythm

The ratio of oesophageal lead DACL to left atrial diameter was higher in patients who remained in sinus rhythm at 6 weeks, 3.5 ± 0.6 ms/mm, than in those who reverted to AF, who had a mean ratio of 3.1 ± 0.4 ms/mm with borderline significance (p = 0.05). A similar difference was seen when calculated based upon lead V1, again with borderline significance (3.6 ± 0.4 vs. 3.2 ± 0.5 ms/mm respectively, p = 0.05). The optimal parameter appeared to be the ratio of the lowest DACL (oesophageal lead or V1) to left atrial diameter, which was significantly higher in patients remaining in sinus rhythm (3.4 ± 0.6 vs. 3.1 ± 0.4 ms/mm respectively, p = 0.04, Figure 3c and Figure 4).

## Discussion

### Electrical and structural atrial remodeling – effect on AF recurrence

During AF alterations of the electrophysiological properties of the atria occur, evidenced by progressive shortening in the refractory periods and loss of normal rate adaptation response[9,25]. Previous studies have shown that a critical number of existing wavelets is essential for AF to be sustained. Since the length of the wavelet is the product of myocardial conduction velocity and refractory period, a decrease in either conduction velocity or a decrease of the refractory period will lead to an increase in the maximum number of wavelets that can be accommodated in a constant area. Similarly, a larger number of circulating wavelets can exist on the surface of larger atria[26,27]. During the recovery period, following conversion to SR, the atria have a heightened predilection of AF recurrence[9,12,25]. Atria exhibiting a short wavelength have been shown to be more vulnerable to arrhythmia induction[24]. This is in agreement with observations that the ease of initiation and probability of maintenance of AF are related to the fibrillatory cycle length[27,28]. The time-course of restoration of normal atrial electrophysiology is poorly understood, but appears to be partially dependent on the duration of the arrhythmia before cardioversion. Studies suggest that atrial effective refractory period reverts to normal within three days of cardioversion of long-standing AF[14]. It has been postulated that electrical remodeling is partially due to intracellular calcium overload since verapamil attenuates the process[11,12,15]. Pre-treatment with verapamil may reduce[6,7,29] and that digoxin[30] delays reversal of electrical remodeling, which may have an impact on the incidence of early AF recurrence. Moreover, AF also leads to progressive mechanical dysfunction[31], seen as post cardioversion atrial stunning. A recent study has shown a relationship between transmitral peak A wave velocity and relapse into AF[32]. Left atrial dilation in patients with AF may be caused by underlying cardiovascular and valvular disease, but progressive dilatation is also a consequence of the arrhythmia itself[33,34]. Conversely, left atrial diameter decreases after successful cardioversion to sinus rhythm[35]. Atrial dilation increases the predilection to AF, and to its post-cardioversion recurrence, probably by impeding intra-atrial conduction and by increasing the atrial area in which multiple wavelet re-entry can occur.

### Prediction of successful restoration of sinus rhythm

Sinus rhythm was restored in 32 of 37 patients. Patients who failed cardioversion tended to have larger left atria and, paradoxically, a longer atrial fibrillatory cycle length. Neither parameter however, nor the combination of the two, proved an important predictor of outcome. Successful cardioversion is dependent upon cardioversion technique and transthoracic impedance, which is influenced by body size, obesity and the presence of pulmonary disease[36]. Some data suggest left atrial enlargement decreases the probability of cardioversion[8] while others do not[37]. Thus although there may be a contribution of electrical and structural remodeling to cardioversion rates, the major determinants are likely to be procedural.

### Prediction of maintaining sinus rhythm post-cardioversion

Previous studies have shown that type of underlying disease, AF duration and the patients functional class are predictors of maintaining sinus rhythm after cardioversion[1,4,5,38]. Radiographic determined heart size[4,5] and increased of the mitral inflow Doppler A-wave amplitude 4 to 24 hours post cardioversion also predict AF recurrence[38]. Some studies have found that increased left atrial diameter is a predictor of AF relapse[8,39], but data is conflicting[1]. Our study found a trend for atrial diameter to be greater in patients who ultimately relapsed back into AF, but this failed to reach statistical significance. Overall these clinical and echocardiographic parameters have low predictive value and a more refined tool is needed.

It is logical that the degree of electrical remodeling will influence the probability of AF recurrence. However, estimation of the degree of remodeling is uncertain because the patients do not have the same initial and final refractory period during AF. Animal studies suggest electrical remodeling develops quickly[11], following a logarithmic curve of rapid initial decline in action potential duration and gradual late decay[9,11], but few data are available regarding the time-course in humans. In addition, the absolute value of the atrial effective refractory period may have an independent influence on AF recurrence as a short refractory period allows the coexistence of more re-entrant wavelets and hence provide a superior substrate for re-initiation. Thus an index of atrial refractory period duration, such DACL, may provide a measure of AF re-inducibility and of the degree of atrial remodeling which is relatively independent of structural and clinical parameters.

The present study found a trend for DACL to be shorter in patients who subsequently relapsed into AF, but this did not prove to be independently predictive. Since all our patients had been in AF for more than a month it is likely that all were fully electrically remodelled, however to different degrees according to the DACL-values. Measurement of DACL may therefore have a more important independent role in predicting arrhythmia recurrence in patients undergoing cardioversion of shorter arrhythmia duration (i.e. AF present for between 1 day and 1 month). In a recent publication[16], the same method as in the present study used to explore the predictive capabilities of an index of atrial refractivity. In the study a significant difference in atrial fibrillatory rate (equals [cycle length]-1) between patients maintaining sinus rhythm for two weeks and those with early AF relapse was demonstrated. The patient characteristics are strikingly similar to the material in the present study with two key exceptions. Firstly, and perhaps most importantly, all patients in the study by Bollmann et al were started on class I or III anti-arrhythmic drug treatment after study inclusion. I.e. patients were investigated without anti-arrhythmic drugs, but during follow-up they were all on anti-arrhythmic drugs, making the interpretation of the finding less obvious. Secondly, in the study by Bollmann et al the mean duration was even longer. Logically, this would make indices of electrical remodeling less good predictors of SR maintenance[40].

The ratio of DACL and left atrial size was the only significant predictor in our primary analysis for the maintenance of sinus rhythm six weeks after DC-cardioversion. In the study of Bollmann et al the predictive properties of atrial fibrillatory rate were improved by mathematically deriving an arbitrary formula including systolic left atrial size. We were unable to validate this formula. To the best of our knowledge, this is the first report, which has combined electrophysiological and anatomical atrial variables, as a pre-specified entity, in a study of outcome following cardioversion. These results are interesting as they assess different aspects of atrial disease, electrical remodeling and dilatation, respectively. However, if these measurements also have any predictive value in regard of more long-term result remains to be evaluated.

### Study limitations

A limitation of our study was that refractory period is estimated indirectly from the dominating atrial cycle length during AF. The method does not allow discrimination of the refractory period from conduction velocity. Thus, the DACL can only be used as an index of the refractory period. Moreover, being an average over time, DACL does not take rapid fluctuations in electrical electrophysiology into account (i.e. rate adaptive response)[41,42]. Utilisation of calcium channel antagonists was not controlled or randomised as it was not known at the time of study design that these agents may reduce early arrhythmia recurrence[7].

Atrial size is assessed by measuring the parasternal anterior-posterior dimension of the left atrium rather than determining atrial area or volume. Failure of the atrial diameter to predict AF recurrence could be due to small sample size. Therefore it remains unclear which measurement of atrial size that best reflects the propensity of the arrhythmia to recur. While this may be hypothetical drawback, a recent study found no benefit of left atrial volume over anterior-posterior diameter in predicting the outcome of cardioversion[43].

## Conclusion

This study demonstrates that combining electrophysiological and anatomical measurements only slightly improves the prediction of maintenance of sinus rhythm following DC-cardioversion of AF. However, the ability to identify those who will relapse using an index of the refractory period during AF failed, underlining that other ways to asses electrical remodeling and / or other variables besides electrical remodeling should be considered in determining the outcome following cardioversion. Documented additional triggers and promoters of AF include ectopic atrial foci[44], interatrial impulse block[45] and premature atrial impulses[46]. Identification and quantification of these may be required to improve risk stratification for arrhythmia recurrence.

## Competing interests

The author(s) declare that they have no competing interests.

## Authors' contributions

CJM participated in the design of the study, included the patients, analysed the data and drafted the manuscript. JEPW, FH and JC analysed and interpreted the data and drafted the manuscript. AR, CJL and MPI acquired data and have been revising the manuscript. MS and LS analysed and interpreted the data and revised the manuscript. SBO designed the study and drafted and revised the manuscript. All authors read and approved the final manuscript.

## Pre-publication history

The pre-publication history for this paper can be accessed here:


